# Developing a FEES service in South Africa: Interdisciplinary reflections for dysphagia care

**DOI:** 10.4102/sajcd.v72i2.1115

**Published:** 2025-11-17

**Authors:** Nancy Barber, Timothy Capon, Marina de Lira de Gouveia, Nolitha Radebe

**Affiliations:** 1 Department of Speech-Language Pathology, Faculty of Humanities, University of the Witwatersrand, Johannesburg, South Africa; 2 Private, Alberton, South Africa

**Keywords:** interdisciplinary assessment, dysphagia, FEES, speech therapy, otolaryngology

## Abstract

**Background:**

Dysphagia is a common and serious condition in critically ill patients, often associated with complications such as aspiration pneumonia and prolonged hospital stays. In South Africa, limited access to instrumental assessments such as fibreoptic endoscopic evaluation of swallowing (FEES) hinders accurate diagnosis and management, particularly in public healthcare settings.

**Objectives:**

This study aimed to critically reflect on the process of implementing an interdisciplinary FEES service in a private South African hospital and to explore how these insights could inform dysphagia care across healthcare sectors.

**Method:**

A duoethnographic approach was used, involving structured reflection among four co-researchers (three speech-language therapists [SLTs] and one otolaryngologist) who co-developed the FEES service. Data were collected through a recorded focus group and analysed using Braun and Clarke’s reflexive thematic analysis. Trustworthiness was ensured through triangulation, member checking, and peer debriefing.

**Results:**

Six themes were identified: (1) catalysts for change in dysphagia management, (2) cultivating interprofessional collaboration, (3) navigating logistical and resource constraints, (4) advocating for FEES, (5) enhancing clinical decision-making, and (6) translating FEES into broader contexts. These themes illustrated how FEES improved diagnostic accuracy, fostered interdisciplinary collaboration, and offered scalable potential for public health systems.

**Conclusion:**

Implementing an interdisciplinary FEES service in a private hospital revealed both challenges and solutions relevant to broader healthcare settings in the South African context.

**Contribution:**

Fibreoptic endoscopic evaluation of swallowing offers a cost-effective, accessible diagnostic option for dysphagia care and should be considered for wider adoption within South Africa’s National Health Insurance framework.

## Introduction

Swallowing is a highly complex physiological process requiring the coordination of multiple brain structures, cranial nerves, and muscles, working in synchrony with respiration to prevent aspiration (Mayerl et al., 2023). Dysphagia, a disruption of this process, is a prevalent condition with serious medical consequences, including aspiration pneumonia, malnutrition, increased hospital stays, and mortality (Likar et al., 2024). In critical care settings, dysphagia frequently results from factors such as direct trauma, neuromyopathy, impaired cognition, tracheostomy placement, and post-extubation complications (Brodsky et al., 2020; Spronk et al., 2022).

In South Africa, resource limitations present significant challenges in dysphagia management. While bedside assessments conducted by speech-language therapists (SLTs) remain the primary method of evaluation, these lack visualisation of the pharynx and larynx, making them less sensitive in detecting conditions such as silent aspiration (Moss et al., 2020). The use of instrumental assessments, particularly fibreoptic endoscopic evaluation of swallowing (FEES), is crucial for enhancing diagnostic accuracy and informing clinical decision-making (Langmore et al., 2022). Fibreoptic endoscopic evaluation of swallowing is performed by inserting a flexible endoscope through the nasal passage towards the larynx to visualise the pharynx, larynx, and subglottis before, during, and after swallowing (Langmore, 2006). This objective, real-time assessment allows clinicians to identify aspiration, residue, and structural or functional impairments, thereby informing individualised diet modifications, therapeutic strategies, and safety measures. However, access to FEES is uneven across the South African healthcare system, as financial and logistical barriers limit its availability, particularly in public hospitals. Given the high patient burden and the need to prevent prolonged hospital stays, optimising dysphagia assessment and management strategies is a priority in both private and public healthcare settings (Spronk et al., 2022).

Interdisciplinary collaboration has been increasingly recognised as essential in dysphagia care, as it allows for a more comprehensive evaluation and integrated management approach (Langmore et al., 2022). While traditional multidisciplinary models involve professionals working separately within their own domains, interdisciplinary approaches encourage joint decision making, leading to more efficient and patient-centred care (Wilkinson et al., 2021). Within an interdisciplinary FEES service, the otolaryngologist (ENT) plays a crucial role in conducting the endoscopic procedure, managing any anatomical or medical complications that may arise. At the same time, the SLT brings specialised expertise in assessing swallowing anatomy and physiology as well as interpreting findings to guide therapy. By working collaboratively during the assessment, sharing clinical observations, co-interpreting findings in real time, and jointly planning patient management, ENTs and SLTs can ensure more accurate diagnoses and coordinated care. Implementing an interdisciplinary FEES service in South Africa has the potential to bridge existing gaps in dysphagia management, particularly if it can be adapted to both private and public healthcare systems.

A growing body of research supports interdisciplinary models for dysphagia assessment, particularly in complex cases where collaboration among SLTs, ENTs, and nursing staff can enhance diagnostic precision and intervention planning (Wilkinson et al., 2021). However, in South Africa, such collaboration remains limited, with dysphagia assessment often occurring in professional silos. This fragmented approach can compromise continuity of care and limit the effectiveness of interventions (Langmore et al., 2022).

Although the benefits of FEES within interdisciplinary practice are well recognised (Langmore et al., 2022), its adoption in South Africa has been inconsistent and largely confined to private healthcare settings. Financial constraints, limited training opportunities, and systemic barriers contribute to the underutilisation of this valuable diagnostic tool (Langmore et al., 2022). Addressing these challenges is essential for ensuring more equitable access to effective dysphagia management across healthcare contexts.

This duoethnographic reflection aimed to critically examine the process of initiating an interdisciplinary FEES service in a private hospital in (city), South Africa. By exploring the practicalities, challenges, and benefits of implementation, the study aimed to generate insights that could inform the integration of interdisciplinary dysphagia care, particularly FEES, into both private and public healthcare systems in South Africa.

## Research methods and design

This study employed duoethnography, a qualitative methodology that facilitates critical reflection through structured dialogue between two or more researchers, enabling the exploration of multiple perspectives and the examination of complex healthcare practices and interdisciplinary collaboration (Norris & Sawyer, 2020). Four co-authors, each directly involved in establishing the FEES service and representing the disciplines SLT (*n* = 3) and ENT (*n* = 1), participated as co-researchers. All authors have received formal training in the use of FEES for dysphagia assessment. N.B. has 18 years of clinical experience, including 8 years conducting FEES; M.d.L.d.G has 6 years of experience, with 3 years in FEES; N.R. has 4 years of clinical experience and 1 year conducting FEES. T.C. is an ENT specialist with 13 years of experience. All authors currently practice in private healthcare but have had public healthcare experience. Despite this private healthcare setting, resource constraints persist because of medical aid limitations and limited equipment. It must also be noticed that even though this is a private setting, the patient population is culturally and linguistically diverse, requiring tailored communication and consideration of diagnoses when explaining the FEES procedure. Ethical approval was obtained from the University of Witwatersrand’s Human Research Ethics Committee (non-medical) (protocol number: H25/03/02), and all participants provided written informed consent.

Data were collected through a structured focus group discussion guided by key research questions reflecting on the implementation of the FEES service. This session was held in a neutral space, recorded, transcribed verbatim, and analysed using Braun and Clarke’s (2021) six-phase reflexive thematic analysis to identify key patterns, shifting perspectives, and implications for dysphagia care in South Africa’s private and public healthcare contexts.

To ensure trustworthiness, the study followed Lincoln and Guba’s (1985) criteria of credibility, transferability, dependability, and confirmability. Credibility was strengthened through the triangulation of perspectives, prolonged engagement, member checking, and peer debriefing. Thick description supported transferability, while dependability was addressed through an audit trail. Confirmability was ensured by grounding interpretations in participants’ voices and incorporating peer review. This systematic and transparent approach ensured that the findings were rigorous, reflective, and applicable across healthcare settings (Hadi & Closs, 2016).

### Ethical considerations

Ethical clearance to conduct this study was obtained from the University of the Witwatersrand’s Human Research Ethics Committee (non-medical) (protocol number: H25/03/02), and all participants provided written informed consent.

## Results

**FIGURE 1:**
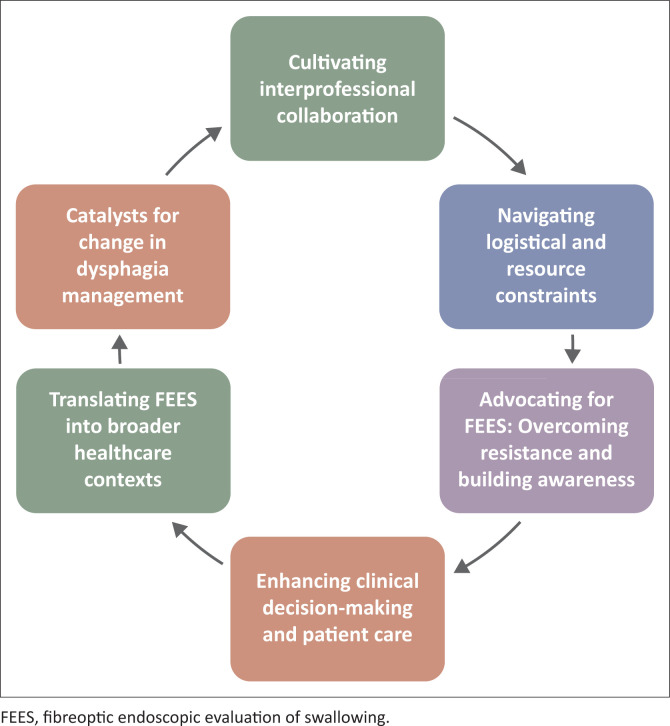
Key themes from the development of an interdisciplinary fibreoptic endoscopic evaluation of swallowing service in a private hospital.

This section presents key themes that emerged from our collaborative reflections on the development of an interdisciplinary FEES service in a private hospital. Through structured discussions, we explored the motivations that drove the initiative, the dynamics of interprofessional collaboration, and the practical and systemic challenges encountered. We also reflected on the clinical and operational impact of FEES, including its potential to enhance patient care, inform public sector practices, and contribute to the broader goals of equitable healthcare within South Africa’s evolving National Health Insurance (NHI) framework. Each theme is presented as an integrated account of our experiences, grounded in critical reflection and shared learning. Figure 1 represents the six themes identified.

### Catalysts for change in dysphagia management

Our motivation for establishing the FEES service within the hospital stemmed from the recurring challenges we encountered with traditional swallowing assessments, such as clinical bedside swallowing evaluations and the use of barium swallows (which were not modified or completed with the assistance of an SLT). This led to the need for more efficient and accurate diagnostic tools. As speech therapists, we frequently faced logistical obstacles when attempting to conduct modified barium swallow assessments, particularly in intensive care units (ICUs), where patients were often immobilised or connected to various monitoring equipment and infusion pumps. In addition, the need to transport patients to different sections in the hospital for these assessments, coupled with long waiting times at the radiology department, created inefficiencies that directly impacted our ability to deliver timely care.

The frustrations arising from these limitations led us to explore alternatives that could offer more flexibility and precision in assessing and managing swallowing disorders. The introduction of FEES represented a viable solution, providing a more immediate, on-site, and accurate assessment method. Our commitment to improving patient care and optimising our time, without compromising the quality of our evaluations, acted as the key catalyst for initiating this change.

### Cultivating interprofessional collaboration

A critical element in the successful implementation of the FEES service was the development of strong interprofessional collaboration. We quickly realised that for this service to be effective, it required seamless cooperation between various departments, particularly between speech therapy and ENT. Our initial discussions with Dr. Capon (ENT) highlighted the importance of clear communication and mutual respect, which were essential for establishing the service. By fostering a shared understanding of each other’s roles and responsibilities, we were able to coordinate efforts and optimise the logistics of implementing FEES in a busy private hospital environment.

Equally important was the involvement of the nursing staff. As we began to integrate FEES into our clinical practice, we found that the nurses’ understanding of their role in the procedure was paramount to its success. We recognised the value of aligning our workflows with the broader hospital team, which required ongoing dialogue and collaboration. By being mindful of each other’s constraints and pressures, we were able to create a more supportive working environment that facilitated the successful delivery of FEES, even amid the hospital’s demanding schedule.

### Navigating logistical and resource constraints

One of the most significant challenges we encountered when developing the FEES service was navigating the logistical and resource limitations of the hospital. Scheduling assessments was often complicated by the high volume of patients and the limited availability of staff and equipment. Early on, we recognised that it would be crucial to establish dedicated time slots for FEES procedures to ensure that they could be conducted without unnecessary delays. The flexibility of our scheduling, as well as our ability to adjust to the busy demands of the hospital, helped to make the service more efficient and sustainable.

Resource constraints also extended to equipment availability. At the time, the FEES service depended on a single video nasopharyngoscope, owned by an ENT colleague, and access to the equipment was not always guaranteed. This occasionally made it challenging to schedule and conduct assessments consistently. However, we worked collaboratively to coordinate the use of the equipment as effectively as possible. These experiences highlighted the value of investing in dedicated resources to support the sustainability of the service and enhance clinical decision-making.

### Advocating for fibreoptic endoscopic evaluation of swallowing: Overcoming resistance and building awareness

Throughout the development of the FEES service, we encountered some resistance, particularly from other healthcare professionals (such as the doctors, specialist physicians and surgeons) who were unfamiliar with the procedure or sceptical about its effectiveness. Initially, some healthcare professionals questioned the necessity of FEES when other diagnostic tools, such as bedside evaluations and barium swallows, had been used for years.

To address this resistance, we focused on advocacy and education. It became clear that we needed to demonstrate the benefits of FEES to other healthcare professionals actively. This involved providing evidence of its accuracy and efficiency in diagnosing swallowing disorders, and highlighting how it could improve patient care. Over time, as more healthcare professionals witnessed the positive impact on patient outcomes, the resistance began to diminish. By consistently emphasising the clinical value of FEES and its role in enhancing treatment planning, we were able to foster broader acceptance within the hospital.

### Enhancing clinical decision-making and patient care

The introduction of FEES profoundly impacted our clinical decision-making and patient management. One of the primary advantages of FEES is its ability to provide objective, real-time data on swallowing function. This has enabled us to make more informed decisions about treatment plans, particularly in patients with complex swallowing issues. With traditional assessment methods, we had to rely on clinical judgement; however, FEES provided us with a direct visual representation of the patient’s condition, allowing us to tailor interventions more precisely.

Furthermore, FEES provided objective insights into whether a feeding gastrostomy tube was indicated or if swallowing exercises could be a viable alternative. For instance, we were able to identify patients who simply needed swallowing exercises rather than a gastrostomy tube, which not only improved patient outcomes but also reduced the associated risks and costs. The ability to make more accurate and timely clinical decisions has been one of the most rewarding aspects of integrating FEES into our practice.

### Translating fibreoptic endoscopic evaluation of swallowing into broader healthcare contexts

The successful implementation of FEES in our hospital has broader implications, particularly for resource-constrained healthcare settings. Despite the logistical challenges we faced, we recognised that FEES offers significant potential for improving patient care, especially in environments where traditional diagnostic tools may be limited or unavailable. By eliminating the need for patient transportation and reducing long wait times for modified barium swallows, FEES can be a more efficient and cost-effective alternative in hospitals with limited resources.

In particular, we see the potential for FEES to be integrated into government healthcare facilities, where there is often a shortage of diagnostic resources. Its relatively low cost, compared to other diagnostic procedures, and its ability to provide immediate feedback make it an attractive option for settings where quick, accurate decision-making is crucial. In addition, we believe that the insights gained from our experience with FEES could inform future healthcare policies, particularly in the context of South Africa’s NHI framework. Demonstrating the value of FEES in improving patient outcomes and reducing healthcare costs could play a significant role in shaping healthcare practices on a national scale.

## Discussion

The initiation of a FEES service within our hospital practice was driven by practical and logistical challenges associated with traditional swallowing assessments, particularly clinical bedside evaluations and standard barium swallow procedures. These challenges are widely acknowledged in the literature, particularly in critical care environments where patient immobility, complex medical conditions, and logistical constraints can significantly limit the utility of standard assessments. Spronk et al. (2022) similarly highlighted global barriers in dysphagia management within ICU settings, including difficulties in transporting patients and delays in accessing diagnostic results. Ponfick et al. (2015) echoed these limitations, underscoring the necessity for diagnostic tools that align with the complexities inherent in critical illness. The introduction of FEES in our hospital effectively addressed these challenges, providing an efficient, precise, and immediately actionable diagnostic alternative at the bedside. The benefits of FEES, particularly its real-time diagnostic capability without radiation exposure, are consistent with the advantages outlined by Langmore (2022).

Interdisciplinary collaboration emerged as a foundational component for the successful implementation and sustainability of the FEES service. The cooperation among speech therapists, ENTs, and nursing staff was essential, facilitating smooth integration and operational efficiency. This collaborative framework aligns with findings from other studies, which emphasise the necessity of multidisciplinary teams in managing complex clinical procedures such as FEES (Spronk et al., 2022; Van Snippenburg et al., 2018). Langmore (2022) further emphasised that effective communication and clear role delineation among team members significantly enhance clinical outcomes in dysphagia management. Our experience reinforced the importance of mutual respect and clear communication in fostering an environment conducive to innovation and quality patient care.

Despite the successful collaboration, our experience highlighted logistical and resource-related challenges. Limited access to the FEES equipment, owned privately by an ENT colleague, introduced some scheduling and operational difficulties. This issue underscores broader themes observed in the literature, particularly regarding the shortage of diagnostic resources and equipment accessibility, common barriers in ICUs worldwide (Spronk et al., 2022). Strategic scheduling and collaborative equipment sharing have alleviated some of these constraints. Our experience reflects the importance of institutional support for diagnostic services, highlighting that investment in dedicated, reliable equipment is crucial for ensuring sustainability and service effectiveness, a sentiment shared by Ponfick et al. (2015).

Initial resistance to adopting FEES within our clinical community also mirrored broader trends noticed internationally. Spronk et al. (2022) and Van Snippenburg et al. (2018) reported similar reluctance among clinicians to embrace newer diagnostic methods, often because of familiarity with established practices or uncertainty regarding new technologies. In addressing this challenge, our proactive educational and advocacy strategies effectively demonstrated FEES’ clinical value, resulting in greater acceptance and integration. Langmore (2022) and Langmore (2017) advocate for comprehensive training and sustained educational initiatives as fundamental in overcoming scepticism and facilitating widespread adoption. Our experience confirms that continuous professional education is vital not only for initial adoption but also for maintaining long-term buy-in and clinical proficiency.

The clinical benefits of incorporating FEES into our diagnostic toolkit were significant and immediate. Fibreoptic endoscopic evaluation of swallowing allowed for detailed visualisation of swallowing function, facilitating more accurate and individualised treatment plans, thus reducing reliance on invasive procedures such as feeding tube placements. Ponfick et al. (2015) reported similar outcomes, observing that FEES significantly reduced unnecessary interventions by providing precise and objective diagnostic information. Langmore (2022) similarly emphasised FEES’ potential in clinical decision-making, underscoring how detailed visual assessment guides more effective treatment planning, thereby enhancing patient safety and reducing healthcare costs. Our practical experience validated these findings, underscoring the potential of FEES to improve both clinical decision-making and patient outcomes.

Finally, the implementation of FEES in our hospital holds significant implications for broader healthcare contexts, especially public healthcare sectors within resource-limited environments. As outlined by Langmore (2022) and Spronk et al. (2022), FEES offers a viable and cost-effective alternative to traditional diagnostic procedures, particularly in resource-constrained settings within our public healthcare system. Given the ongoing reforms towards the NHI system in South Africa, our insights offer practical lessons on how FEES could be effectively integrated into government-run healthcare facilities. Demonstrating FEES’ cost-effectiveness, ease of use, and clinical efficacy could significantly influence healthcare policies in the NHI, thereby improving access to dysphagia assessment and management services and improving patient outcomes nationwide.

### Limitations

As the duoethnography methodology is subjective and based on the lived experiences of the researchers, the accounts presented in this study may potentially introduce bias from the researchers, as their accounts are subjective and limited to their perspectives (Sung et al., 2023). Furthermore, as the researchers’ experiences are the focus of this study, they may have limited confidentiality and could have censored their accounts because of concerns of being ostracised by their colleagues (Burleigh & Burm, 2022; Sung et al., 2023). The credibility of this study was, however, upheld by reducing bias through peer debriefing as peer researchers provided their input to improve the quality of the research study (Enworo, 2023).

## Conclusion

In conclusion, our experience in developing an interdisciplinary FEES service demonstrates clear advantages in clinical effectiveness, interdisciplinary collaboration, resource optimisation, and potential healthcare policy implications. Overcoming initial barriers through strategic planning, professional education, and interdisciplinary collaboration has proven critical in realising FEES’ full potential. Future research should explore long-term clinical outcomes, cost-benefit analyses, and broader integration strategies, especially within the evolving NHI framework in South Africa.

## References

[CIT0001] Braun, V., & Clarke, V. (2021). Can I use TA? Should I use TA? Should I not use TA? Comparing reflexive thematic analysis and other pattern-based qualitative analytic approaches. *Counselling and Psychotherapy Research*, 21(1), 37–47.https://doi.org/10.1002/capr.12360

[CIT0002] Brodsky, M.B., Pandian, V., & Needham, D.M. (2020). Post-extubation dysphagia: A problem needing multidisciplinary efforts. *Intensive Care Medicine*, 46(1), 93–96. 10.1007/s00134-019-05865-x 31768568 PMC7219527

[CIT0003] Burleigh, D., & Burm, S. (2022). Doing duoethnography: Addressing essential methodological questions. *International Journal of Qualitative Methods*, 21, 1–8. 10.1177/16094069221140876

[CIT0004] Enworo, O.C. (2023). Application of Guba and Lincoln’s parallel criteria to assess trustworthiness of qualitative research on indigenous social protection systems. *Qualitative Research Journal*, 23(4), 372–384. 10.1108/QRJ-08-2022-0116

[CIT0005] Hadi, M.A., & Closs, S.J. (2016). Ensuring rigour and trustworthiness of qualitative research in clinical pharmacy. *International Journal of Clinical Pharmacy*, 38(3), 641–646. 10.1007/s11096-015-0237-6 26666909

[CIT0006] Langmore, S.E. (2006). *Endoscopic evaluation of oral and pharyngeal phases of swallowing*. GI Motility Online.

[CIT0007] Langmore, S.E. (2017). History of fiberoptic endoscopic evaluation of swallowing for evaluation and management of pharyngeal dysphagia: Changes over the years. *Dysphagia*, 32(1), 27–38. 10.1007/s00455-016-9775-x 28101663

[CIT0008] Langmore, S.E., Scarborough, D.R., Kelchner, L.N., Swigert, N.B., Murray, J., Reece, S., Cavanagh, T., Harrigan, L.C., Scheel, R., Gosa, M.M., & Rule, D.K. (2022). Tutorial on clinical practice for use of the fiberoptic endoscopic evaluation of swallowing procedure with adult populations: Part 1. *American Journal of Speech-Language Pathology*, 31(1), 163–187. 10.1044/2021_AJSLP-20-00348 34818509

[CIT0009] Likar, R., Aroyo, I., Bangert, K., Degen, B., Dziewas, R., Galvan, O., Grundschober, M.T., Köstenberger, M., Muhle, P., Schefold, J.C., & Zuercher, P. (2024). Management of swallowing disorders in ICU patients – A multinational expert opinion. *Journal of Critical Care*, 79, 154447. 10.1016/j.jcrc.2023.154447 37924574

[CIT0010] Lincoln, Y.S., & Guba, E.G. (1985). *Naturalistic inquiry*. Sage.

[CIT0011] Mayerl, C.J., Gould, F.D.H., Adjerid, K., Edmonds, C., & German, R.Z. (2023). The pathway from anatomy and physiology to diagnosis: A developmental perspective on swallowing and dysphagia. *Dysphagia*, 38(1), 33–41. 10.1007/s00455-022-10449-x 35441265 PMC9579268

[CIT0012] Moss, M., White, S.D., Warner, H., Dvorkin, D., Fink, D., Gomez-Taborda, S., Higgins, C., Krisciunas, G.P., Levitt, J.E., McKeehan, J., McNally, E., Rubio, A., Scheel, R., Siner, J.M., Vojnik, R., & Langmore, S.E. (2020). Development of an accurate bedside swallowing evaluation decision tree algorithm for detecting aspiration in acute respiratory failure survivors. *Chest*, 158(5), 1923–1933. 10.1016/j.chest.2020.07.051 32721404 PMC7674978

[CIT0013] Norris, J.J., & Sawyer, R.D. (2020). Duoethnography: A polytheoretical approach to (re)storing, (re)storying the meanings that one gives. In P. Leavy (Ed.), *The Oxford handbook of qualitative research* (2nd ed., pp. 397–423). Oxford University Press.

[CIT0014] Ponfick, M., Linden, R., & Nowak, D.A. (2015). Dysphagia – A common, transient symptom in critical illness polyneuropathy: A fiberoptic endoscopic evaluation of swallowing study. *Critical Care Medicine*, 43(2), 365–372. 10.1097/CCM.0000000000000705 25377021

[CIT0015] Spronk, P.E., Spronk, L.E., Egerod, I., McGaughey, J., McRae, J., Rose, L., & Brodsky, M.B. (2022). Dysphagia in intensive care evaluation (DICE): An international cross-sectional survey. *Dysphagia*, 37(6), 1451–1460. 10.1007/s00455-021-10389-y 35092486

[CIT0016] Sung, R.J., Holt, E.A., & Lo, S.M. (2023). Constructing biology education research identities: A duoethnography. *Frontiers in Education*, 8, 1134040.

[CIT0017] Van Snippenburg, W., Kröner, A., Flim, M., Hofhuis, J., Buise, M., Hemler, R., & Spronk, P. (2019). Awareness and management of dysphagia in Dutch intensive care units: A nationwide survey. *Dysphagia*, 34, 220–228 30069599 10.1007/s00455-018-9930-7

[CIT0018] Wilkinson, J.M., Codipilly, D.C., & Wilfahrt, R.P. (2021). Dysphagia: Evaluation and collaborative management. *American Family Physician*, 103(2), 97–106. 33448766

